# Pressurized natural deep eutectic solvents: An alternative approach to agro-soy by-products

**DOI:** 10.3389/fnut.2022.953169

**Published:** 2022-09-08

**Authors:** Felipe Sanchez Bragagnolo, Bárbara Socas-Rodríguez, Jose A. Mendiola, Alejandro Cifuentes, Cristiano Soleo Funari, Elena Ibáñez

**Affiliations:** ^1^Green Biotech Network, School of Agricultural Sciences, São Paulo State University, Botucatu, Brazil; ^2^Laboratory of Foodomics, Institute of Food Science Research, CIAL, CSIC-UAM, Madrid, Spain

**Keywords:** green chemistry, bioactive compounds, bioeconomy, biorefinery, NADES, soy by-products, pressurized liquid

## Abstract

Soybeans are mainly used for food and biodiesel production. It is estimated that soy crops worldwide will leave about 651 million metric tons of branches, leaves, pods, and roots on the ground post-harvesting in 2022/23. These by-products might serve as largely available and cheap source of high added-value metabolites, such as flavonoids, isoflavonoids, and other phenolic compounds. This work aimed to explore green approaches based on the use of pressurized and gas expanded-liquid extraction combined with natural deep eutectic solvents (NADESs) to achieve phenolic-rich extracts from soy by-products. The total phenolic and flavonoid contents of the generated extracts were quantified and compared with conventional solvents and techniques. Pressurized liquid extraction (PLE) with choline chloride/citric acid/water (1:1:11 – molar ratio) at 120^°^C, 100 bar, and 20 min, resulted in an optimized condition to generate phenolic and flavonoid-rich fractions of soy by-products. The individual parts of soy were extracted under these conditions, with their metabolic profile obtained by UHPLC-ESI-QToF-MS/MS and potential antioxidant properties by ROS scavenging capacity. Extracts of soy roots presented the highest antioxidant capacity (207.48 ± 40.23 mg AA/g), three times higher than soybean extracts (68.96 ± 12.30). Furthermore, Hansen solubility parameters (HSPs) were applied to select natural hydrophobic deep eutectic solvents (NaHDES) as substituents for *n*-heptane to defat soybeans. Extractions applying NaHDES candidates achieved a similar yield and chromatography profile (GC-QToF-MS) to *n*-heptane extracts.

## Introduction

Compounds identified in natural products have been historically used to develop new drugs, ingredients for functional foods, cosmetic products, and other applications (1). Commodity crops, such as apples, coffee, olives, and soybeans, contain several metabolites with potential bioactivity (2–5). Soybeans, the major oilseed crop worldwide, present a wide range of phenolic compounds, mainly isoflavonoids and flavonoids, resulting in the growing interest for being more than just an oilseed plant (5). Genistin, an isoflavone glycoside, contains anti-adipogenic, and anti-lipogenic *in vitro* properties and also prevents breast cancer (6, 7). Apigenin, a flavonoid, has protective effects against cardiometabolic diseases, and it has been suggested as biopesticide (8, 9). In addition, other bioactive metabolites identified in agro-soy by-products collected after harvesting (i.e., branches, leaves, pods, and roots) provide new findings and perspectives for using such materials (10, 11). Soybeans, the soy commodity, have been the second source of vegetable oils, with an estimated production of 61 million tons in 2022/23 (12). The defatting process of soybeans has been widely performed using hexane, a toxic and flammable solvent (13). Alternative solvents, such as ethanol, supercritical CO_2_, and 2-methyloxolane, have already demonstrated to be feasible and greener options to replace the conventional one (13–15). Furthermore, new alternatives as natural hydrophobic deep eutectic solvents (NaHDES) may be promising bio-based solvents for defatting procedures. Moreover, an *in silico* approach applying Hansen solubility parameters (HSPs) can be performed to select the most similar solvent to hexane. Based on the concepts of solubility and “like dissolves like,” HSPs promote the comparison among several solvents and generate a computational simulation of their interaction (16). Solvents with similar HSP could present a higher similarity between their chemical and/or physical properties, meaning a valuable tool to replace conventional and hazardous solvents with sustainable ones.

In this context, green analytical chemistry should be employed to add value to agro-soy by-products, reducing potential environmental problems related to their extraction and revalorization, as suggested by the goal 12, “Sustainable consumption and production” of the 17 sustainable development goals (SDGs) set by the United Nations (UN) (17). Therefore, greener solvents and techniques are required in accordance with a sustainable procedure, reducing energy, reagents, and increasing the security of the applied analytical methodology. Natural deep eutectic solvents (NADESs), which are composed of major metabolites identified in plants and other organisms, could be green alternatives to extract bioactive compounds (18). Duru et al. performed NADES extraction of isoflavones from kudzu root and soy molasses wastes, achieving fractions with higher antioxidant activity and stability than methanolic extraction (19).

To achieve a greener process, the integration of green solvents with innovative and environmentally friendly technologies can offer some advantages such as time and energy reduction, as well as solvent consumption. In this sense, pressurized liquid extraction (PLE) has shown significant benefits for extracting phenolics and other bioactive compounds from natural matrices, despite being scarcely integrated with NADES as solvents (20–23). Nevertheless, this combination was successfully performed to extract anthocyanins from a Brazilian berry processing by-product (24), as demonstrated by Benvenutti et al. (23). In addition, gas-expanded liquids (GXLs), a promising type of solvents, could be useful to decrease the viscosity of NADES, with the addition of CO_2_ at large amounts. Moreover, GXLs promote other modifications in the physicochemical properties of the solvents (24).

Therefore, this work aimed to develop an alternative approach to generate phenolic-rich fractions from soy by-products, i.e., branches, leaves, pods, and roots collected just after mechanical harvesting, combining PLE and GXLs with NADES. For this, (i) an *in silico* approach using HSP was applied to select an alternative NaHDES candidate to replace *n*-heptane in the defatting process, (ii) PLE and GXLs were performed with choline chloride/citric acid/water 1:1:11 (molar ratio) (ChCl:Ca:H2O) to generate phenolic-rich extracts, (iii) the optimized extraction condition was applied to extract the individual parts of agro-soy by-products and soybeans, (iv) the potential reactive oxygen species (ROS) scavenging capacity of the generated extracts was measured, and (v) the metabolite profiling was studied by ultra-high-performance liquid chromatography coupled to high-resolution mass spectrometry (UHPLC-QToF-MS/MS).

## Materials and methods

### Chemicals

High-performance liquid chromatography (HPLC)-grade solvents such as acetonitrile, n-heptane, and ethanol (EtOH), as well as menthol, eucalyptol, thymol, choline chloride, and citric acid were purchased from VWR Chemicals (Spain). Standards of 2,2′-azino-bis (3-ethylbenzothiazoline-6-sulphonic acid) (ABTS), sodium carbonate (Na_2_CO_3_), aluminum chloride (AlCl_3_), potassium persulfate (K_2_S_2_O_8_), disodium phosphate (Na_2_HPO_4_), monopotassium phosphate (KH_2_PO_4_), fluorescein sodium salt, gallic acid, ascorbic acid, and quercetin were obtained from Sigma-Aldrich (Spain). Folin–Ciocalteu phenol reagent was acquired from Merck (Germany). 2,2-azobis (2-amidinopropane) dihydrochloride (AAPH) was purchased from TCI Chemicals (Japan). Ultrapure water was obtained from a Millipore system (Billerica, United States).

### Plant material

Soy branches, leaves, pods, roots, and soybeans (cultivar NA5909) were collected after the mechanical harvesting at the School of Agricultural Sciences of São Paulo State University—Botucatu, São Paulo State, Brazil (−22.8296354, −48.42553). All parts were grounded in a basic analytical mill (IKA^®^ A11, Germany) and separated by granulometry on an electromagnetic sieve shaker (Bertel, Brazil). Only particles with < 0.25 mm were used for extraction.

### Pressurized liquid extraction

The PLEs were made in an ASE 200 device (Dionex, United States) using an 11-ml stainless steel cell at 100 bars. PLE was performed in two steps: (i) a first step in which materials with high content of lipids (soybeans) are defatted, and (ii) a second step focused on phenolic extraction. It is worth mentioning that, for those vegetal materials with low lipid content, the PLE extraction begins on the second step.

**FIGURE 1:**
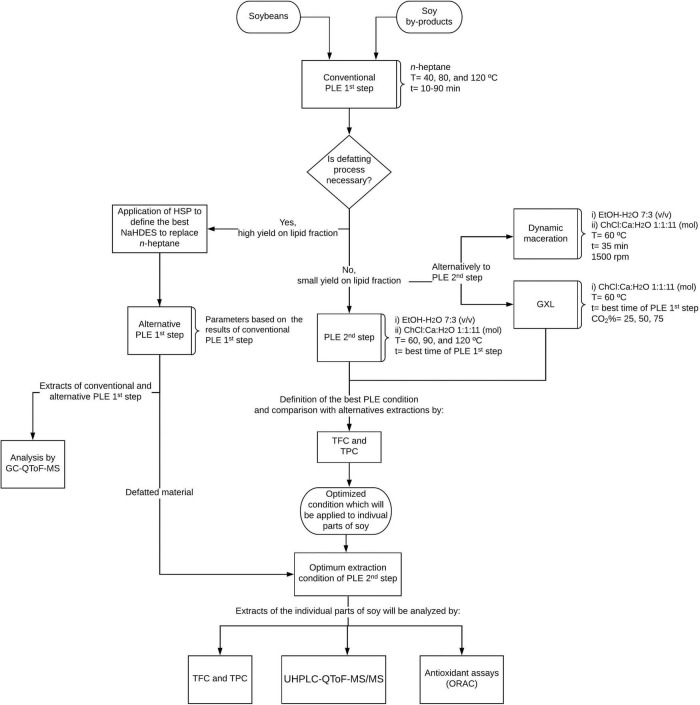
Flowchart of the extraction procedures and analyses employed in this work.

Soy material was separated into the following: (i) a mix of equal parts of soy by-products (250 mg of branches, leaves, pods, and roots—in a total of 1 g) and (ii) 1 g of soybeans (reference material). The vegetal material was mixed with sea sand in a 1:2 w/w proportion, which facilitated the diffusion of the solvent in the material, while avoiding preferential paths. All the extractions were made in triplicate, and the samples were preserved at −18^°^C until further analysis. The flowchart of the extraction processes with the analyses employed in this work is shown in Figure 1.

### Pressurized liquid extraction first step—Defatting procedure

First, a defatting process was performed using *n*-heptane as the benchmark solvent, which is greener but highly similar to hexane regarding the HSP, the conventional solvent used to defat soybeans (25). The extraction procedure was performed at three temperatures (40, 80, and 120^°^C) for 90 min, generating kinetic extraction curves from sample collection each 10 min. The accumulated extraction yield (%) of each extraction was measured by concentrating each extract with a continuous flow of nitrogen gas. To replace *n*-heptane, three NaHDES candidates were selected using HSPs, as well as considering the toxicity and availability of their components (26, 27). The HSPs of 108 NaHDES candidates, which have already been described in the literature, were calculated using the HSPiP^®^ software v 5.0 (28–45). Therefore, the three parameters of HSP, δD, δP, and δH, which correspond to dispersion bonds, dipolar intermolecular force, and hydrogen bonds between molecules/solvents, were estimated for each NaHDES mixture (16). Additionally, Jchem (JChem for Excel 21.1.0.787, ChemAxon^1^ and Microsoft Excel 365 (Microsoft Corporation, United States) were applied to calculate the volume percentage of each compound of NaHDES, as well as to generate their SMILES. Then, the HSPs of the individual components of NaHDES were estimated by the “Do It Yourself” tool, and the final HSPs of NaHDES mixtures were achieved using the “Solvent Optimizer” tool in HSPiP^®^ software v 5.0 (27). The selection of NaHDES was based on the distance (Ra—Equation 1) to *n*-heptane (considered as “solute” in Equation 1). Supplementary Table 1 summarizes the estimated HSP of listed NaHDES and *n*-heptane, as well as the distance between them. Thus, the selected NaHDESs were prepared by mixing their components with a magnetic stirring bar at 90^°^C until a clear liquid was reached (46). The alternative PLE first step using NaHDES was tested at three temperatures (90, 120, and 150^°^C) for 20 min (two extractions of 10 min each). Bottles with NaHDES were kept at 90^°^C in all extractions (Supplementary Figure 1). The resulting extracts were concentrated by a continuous flow of nitrogen gas until dryness.

Equation 1

$$R_{a}=\sqrt{\begin{array}{c} 4\,{\left(\delta \,{\text{D}}^{s\,o\,l\,v\,e\,n\,t}-\delta \,{\text{D}}^{s\,o\,l\,u\,t\,e}\right)}^{2}+{\left(\delta \,{\text{P}}^{s\,o\,l\,v\,e\,n\,t}-\delta \,{\text{P}}^{s\,o\,l\,u\,t\,e}\right)}^{2}\\ +{\left(\delta \,{\text{H}}^{s\,o\,l\,v\,e\,n\,t}-\delta \,{\text{H}}^{s\,o\,l\,u\,t\,e}\right)}^{2} \end{array}}$$

### Pressurized liquid extraction second step—Phenolic extraction

The second step of PLE was first performed and optimized with the mix of soy by-products. Subsequently, the optimal condition was applied to extract the soy by-products individually, as well as the defatted soybean. Hence, two solvents were tested in this step: (i) EtOH-H_2_O 7:3 (v/v), and (ii) choline chloride/citric acid/water 1:1:11 (molar ratio) (ChCl:Ca:H_2_O), which has previously been applied and its constituents and ratio optimized to extract isoflavones and solubilize flavonoids (47, 48). ChCl:Ca:H_2_O was prepared as described in the previous section. Extractions were performed at three temperatures (60, 90, and 120^°^C) for 20 min (two extractions of 10 min each). Subsequently, the condition with the highest phenolic and flavonoid content was applied to extract the soy parts individually.

### Conventional and alternative extraction of agro-soy by-products

Dynamic maceration (Mac) and gas-expanded liquids (GXLs) were performed to extract the mix of agro-soy by-products and compare their total phenolic and flavonoid contents with PLE. The results were evaluated by analysis of variance (ANOVA) and Tukey’s multiple comparisons test with a 5% level of significance on GraphPad Prism version 9 (GraphPad Software, United States).

### Dynamic maceration as the reference extraction technique

Dynamic maceration (Mac) with magnetic stirring was selected as the conventional extraction technique (11). A mix of 100 mg of branches, leaves, pods, and roots—in a total of 400 mg—was extracted with 8 ml of (i) EtOH-H_2_O 7:3 (v/v), and (ii) NADES choline chloride/citric acid/water 1:1:11 (molar ratio—ChCl:Ca:H_2_O), at 1,500 rpm, 60^°^C for 35 min (IKA C-MAG HS 7, Germany).

### Gas-expanded liquids

As an alternative technique, gas-expanded liquid (GXLs) extraction was combined with NADES to achieve a new methodology for phenolic extraction. To the best of our knowledge, the literature does not report such combination. Thus, a homemade compressed fluid extractor was used for gas-expanded liquid (GXLs) extractions. The equipment consisted of a PU-2080 Plus CO_2_ high-pressure pump (Jasco, Japan) and an Agilent 1,200 Binary Pump (Agilent, United States). For the extractions, 1 g of the mix of soy by-products (prepared as stated above) was mixed with 2 g of sea sand and added into a 20-ml stainless-steel extraction cell with glass wool packed at both ends. The cell was placed in an Adept CE 4600 column oven (Cecil, United Kingdom) at 60^°^C. The pressure was kept at 100 bar using a manual metering valve (Swagelok, United States) (49). The total flow rate was established at 4 ml/min, and 25, 50, and 75% CO_2_ in ChCl:Ca:H_2_O were tested. All the extractions were made in triplicate, and the samples were preserved at −18^°^C until further analysis.

### Determination of total phenolic content

Total phenolic content (TPC) was determined by Folin-Ciocalteu method (50). First, 10 μl of extracts was mixed with 600 μl of ultrapure water and reacted with 50 μl of the reagent Folin-Ciocalteu. After 1 min, 150 μl of 20% (w/v) sodium carbonate (Na_2_CO_3_) was added, and the final volume was adjusted to 1 ml with water. The sample was mixed and incubated for 2 h at room temperature in darkness. Subsequently, 300 μl of the mixture was transferred to a 96-well microplate spectrophotometer reader (Synergy HT, United States), and the absorbance was measured at 760 nm. The values were converted to mg GAE (gallic acid equivalent) in the extracts by a calibration curve (0–2 mg/ml) prepared with standard gallic acid. Results were expressed in mg equivalents of gallic acid per g of vegetal material.

### Determination of total flavonoid content

Total flavonoid content (TFC) was measured using the aluminum chloride colorimetric method with some modifications (51). First, 240 μl of methanolic solutions of quercetin (4–14 μg/ml) was used to prepare the calibration curve, and 100 μl of extracts adding 60 μl of AlCl_3_ 8 mM was filled into each well. The absorbance of each sample was measured at 425 nm after 30 min of incubation in darkness. The values were converted to mg QE (quercetin equivalent) in the extracts. Results were expressed in μg equivalents of quercetin per g of vegetal material.

### Antioxidant activity assays—Reactive oxygen species scavenging capacity

The oxygen radical absorbance capacity (ORAC) method was carried out according to Ou et al. (52) and Ninfali et al. (53). Reaction mixtures in the wells contained the following reagents: 100 μl of extract sample at different concentrations (5 μg–50 μg/ml) in EtOH-H_2_O 9:1 (v/v), 100 μl of AAPH (590 mM) in 30 mM phosphate-buffered saline (PBS) at pH = 7.5, 25 μl of fluorescein (10 μM) in PBS buffer, and 100 μl of PBS buffer. Fluorescence was measured (λexcitation = 485 nm; λemission = 530 nm) every 5 min at 37^°^C for 1 h. Ascorbic acid was used as the reference standard. The capacity of each extract for scavenging peroxyl radicals was calculated using Equation 2, where *As* is the area under the curve (AUC) of fluorescein in the sample, *At* is the AUC of the ascorbic acid, *Ab* is the AUC of the control (blank of the extraction), *k* is the dilution factor, *a* is the concentration of the ascorbic acid in mg/ml, and *h* is the ratio between the grams of extract and the grams of plant material used for the extraction (mg AA/g).

Equation 2

$$O\,R\,A\,C\,\left(m\,g\,a\,s\,c\,o\,r\,b\,i\,c\,a\,c\,i\,d\,e\,q\,u\,i\,v\,a\,l\,e\,n\,t/g\right)=\left[\frac{A\,s-A\,b}{A\,t-A\,b}\right]\,k\,a\,h$$

### Gas chromatography coupled to mass spectrometry

Metabolite profiling of soybean *n*-heptane and NaHDES extracts (10 mg/ml) was performed using a 7890B Agilent system (Agilent Technologies, United States) coupled to a quadrupole time-of-flight (QToF) 7200 (Agilent Technologies, United States) equipped with an electronic ionization (EI) interface. Separations were achieved in a 30 m × 250μm × 0.25μm DB5- MS + 10 m Duragard Capillary Column (Zorbax, Agilent Technologies, United States). The samples were diluted at 1:10 in *n*-heptane and filtered with a 0.22-μm nylon syringe filter before injection. The injection volume was 1μl using a split flow of 8.4 ml/min. The helium flow rate was 0.8 ml/min. The injector temperature was 250^°^C. Oven temperature started at 60^°^C (1 min), followed by 325^°^C at the rate of 10^°^C°/min (10 min) and 11-min solvent delay. Metabolites present in the *n*-heptane and eucalyptol/menthol (1:1) extracts were annotated using the match of mass spectra in the Agilent Mass Hunter Unknown Analysis tool and mass spectral databases (i.e., NIST MS Search v.2.0 and Fiehn Lib).

### Ultra-high-performance liquid chromatography coupled to mass spectrometry

Extracts of the individual parts of soy, i.e., branches, leaves, pods, roots, and soybeans, from the optimal condition of PLE (ChCl:Ca:H_2_O at 120^°^C), which presented the highest TPC and TFC), were diluted 1:10 in EtOH-H_2_O 7:3 (v/v) and filtered with a 0.22-μm nylon syringe filter before injection. Analyses were performed in an Agilent 1290 UHPLC system (Agilent Technologies, United States) coupled to an Agilent 6540 quadrupole-time-of-flight mass spectrometer (QToF-MS), equipped with an orthogonal ESI source (Agilent Jet Stream, United States). The analysis in positive and negative modes was performed using a C18 column, 100 mm × 2.1 mm; 1.8μm (Zorbax Eclipse Plus, Agilent Technologies, United States) and H_2_O and ACN, both acidified with 0.01% formic acid (v/v) (A and B, respectively) in the following gradient elution: 0–30% B in 0–7 min, 30–80% B in 7–9 min, 80–100% B in 9–11 min, 100% B in 11–13 min, and 0% B in 13–14 min. The flow rate was kept at 0.5 ml/min, the temperature of the column was 30^°^C, and the sample injection volume was 5μl. MS parameters were as follows: capillary voltage, 4,000 V; nebulizer pressure, 40 psi; drying gas flow rate, 10 L/min; gas temperature, 350^°^C; skimmer voltage, 45 V; fragmentor voltage, 110 V. The MS and auto-MS/MS modes were set to acquire m/z values ranging between 50 and 1,100 and 50–800, respectively, at a scan rate of 5 spectra per second. Agilent Mass Hunter Qualitative Analysis software (B.07.00) was used for post-acquisition data processing. Metabolites present in the extracts were annotated using Global Natural Product Social Molecular Networking (GNPS)^2^ (54). First, MS data were converted to mzML format with ProteoWizard 3.0.6002 package MSConvert software (ProteoWizard, United States). The converted files were uploaded to GNPS platform, and a molecular network was created using the online workflow.^3^ The data were filtered by removing all MS/MS fragment ions within ± 17 Da of the precursor m/z. MS/MS spectra were window filtered by choosing only the top six fragment ions in the ± 50Da window throughout the spectrum. The precursor ion mass and MS/MS fragment ion tolerances were set to 0.02 Da. A cosine score above 0.65 and more than four matched peaks were used to create the edges of the network. The spectra in the network were searched against GNPS’ spectral libraries. The library spectra were filtered in the same manner as the input data. The product ion spectra presented in GNPS was manually verified with previous literature annotation. In addition, isoflavonoids and flavonoids which have already been identified in soy have their *m/z* and MS/MS spectra searched in the chromatograms using MZmine 2.53 software^4^ (55, 56).

## Results and discussion

### Pressurized liquid extraction first step—Defatting soy with n-heptane and alternative natural hydrophobic deep eutectic solvents

**FIGURE 2:**
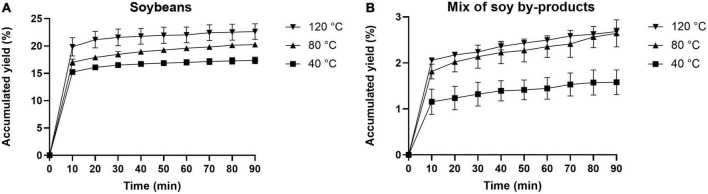
Kinetic curves based on the yields of soybean and mix of soy by-products extracts generated by PLE using *n*-heptane at three different temperatures (40, 80, and 120^°^C).

The kinetic curves corresponding to the first step of PLE of soy (including soybeans and the mix of soy by-products) are shown in Figure 2. Such curves are related to the accumulated yield (%) of the defatting procedure of the soybeans (reference material - A) and of the mix of soy by-products (branches, leaves, pods, and roots - B) using *n*-heptane as the reference solvent. The extraction of soybeans reached equilibrium after 20 min of extraction with an accumulated yield of 21.14% at 120^°^C (Figure 2A). On the other hand, the defatting process for the mix of soy by-products resulted in an accumulated yield lower than 3% for all monitored temperatures and extraction times. Therefore, defatting was not applied to the mix and individual parts of soy by-products since it would involve an additional step with more solvent and energy consumption with a small benefit in terms of oil yield.

As an alternative to *n*-heptane, 108 candidates of NaHDESs, which have already been described in the literature, were screened *in silico* to defat soybeans (28–45). In contrast to the usual approach to HSPs, when the target is a bioactive compound, this work used a solvent, *n*-heptane, as a target to find other similar (and greener) options. The HSP approach was applied using HSPiP^®^ software, allowing the calculation of HSP for NaHDES. A lower distance (Ra) between NaHDES and *n*-heptane means a higher similarity between them and a potential replacer to the benchmarking solvent. Supplementary Table 1 presents the HSP and Ra values of NaHDES, and Table 1 contains the referred values of the three selected NaHDES and *n*-heptane. Eucalyptol/menthol 1:1 (EM) presented a higher similarity to *n*-heptane, with a Ra of 6.33. Such NaHDES candidate has already been applied to extract astaxanthin from brown crab shell residues (45). EM was the least polar and viscous terpene-based mixture in quoted work. The second NaHDES candidate selected was camphor/menthol (1:1) (CM) as it contains one of the same constituents as the first, menthol, and similar Ra (5th place on Supplementary Table 1—Ra of 7.13). It reduces the requirement of the other two substances and contains a percentage difference of 3.63% from the second-ranked NaHDES, borneol/oleic acid (1:4). Camphor/thymol (3:2) was the third mixture chosen as it presents one of the same constituents of the latter mixture and contains intermediate properties among the other two proportions (1:1 and 2:1) of its components.

**TABLE 1:** Hansen solubility parameters (δD, δP, and δH) of *n*-heptane, the reference solvent, and the three selected natural hydrophobic deep eutectic solvents (NaHDES).

Solvents	δD	δP	δH	Ra
*n*-Heptane (reference)	15.30	0.00	0.00	0.00
Eucalyptol:menthol 1:1	16.59	3.17	4.82	6.33
Camphor:menthol 1:1	16.91	4.28	4.71	7.13
Camphor:thymol 3:2	17.56	4.72	4.16	7.74

Then, the three selected NaHDES candidates were initially tested to defat soybean using PLE at 90, 120, and 150^°^C. The temperature of 120^°^C was set as the intermediate temperature since it was the best temperature for *n*-heptane. The achieved yields for the three mixtures were similar to those observed for *n*-heptane, with an accumulated yield of 22.34 ± 1.43% (Table 2 and Supplementary Figure 2). The extraction time was fixed at 20 min, resulting in the equilibrium of the accumulated yield with the reference solvent, as previously described. No statistical differences (*p* > 0.05) were observed for the three mixtures at the three temperatures. It means that extractions with NaHDES candidates at 90^°^C could be successfully used to replace *n*-heptane to defat soybean at 120^°^C. Similar results were achieved by Claux et al. (15). In their work, soybean defatting was performed using dry and aqueous 2-methyloxolane (2-MeTHF), resulting in a similar accumulated yield (23.7 ± 0.1) to our results. For the following steps, EM was selected as the best mixture to defat soybean as it presents similar yields compared to the other options and their components showed lower toxicity (57–60). Gasparetto et al. highlighted green alternatives to extract soybean oil (61). Solvents, such as terpenes, cyclopentyl methyl ether (CPME), and 2-MeTHF, have been applied as suitable replacers for hexane. Moreover, mixtures of ethanol with alkyl ester or ethyl acetate, as well as α-pinene were studied to defat soybean, achieving promising results (62–64). In addition to the HSP, COnductor-like Screening MOdel for Real Solvents (COSMO-RS) can also be employed for screening greener solvents for defatting process, as described by Sicaire et al. (65, 66) and Cascan et al. (67).

**TABLE 2:** Extraction yield of the defatting process of soybeans employing pressurized liquid extraction (PLE) with *n*-heptane at 40, 80, and 120°C and three selected NaHDES candidates, eucalyptol/menthol (1:1), camphor/menthol (1:1), camphor/thymol (3:2), at 90, 120, and 150°C.

Solvents	Temperature (^°^C)	Extraction yield (%)
*n*-Heptane	40	16.08 ± 0.12 ^e^
	80	17.91 ± 0.49 ^d,e^
	120	21.15 ± 1.51 ^b,c^
Eucalyptol:menthol (1:1)	90	21.54 ± 1.53 ^a,b,c^
	120	22.12 ± 0.93 ^a,b,c^
	150	21.79 ± 0.63 ^a,b,c^
Camphor:menthol (1:1)	90	20.32 ± 0.86 ^c,d^
	120	22.62 ± 0.1 ^a,b,c^
	150	23.97 ± 0.55 ^a^
Camphor:thymol (3:2)	90	21.32 ± 1.37 ^a,b,c^
	120	23.51 ± 1.02 ^a,b^
	150	23.84 ± 0.55 ^a,b^

Letters in a column indicate significant differences between extraction solvents for different extracts (p < 0.05) by Tukey’s test.

**FIGURE 3:**
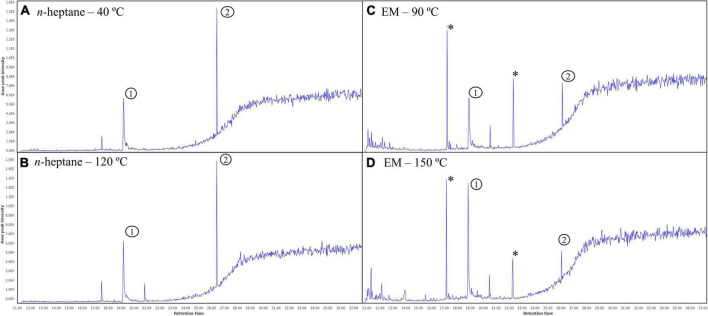
GC-QToF-MS chromatograms of the lower and higher temperature of soybean *n*-heptane extracts at 40 **(A)** and 120^°^C **(B)** and eucalyptol/menthol (1:1) – EM at 90 **(C)** and 150^°^C **(D)** performed by PLE. The peaks marked with * were related to phthalate contaminants identified in NaHDES candidate extracts.

Additionally, Figure 3 shows the GC-QToF-MS chromatograms of the fractions corresponding to the lower and higher temperature of *n*-heptane (40 and 120^°^C) and EM (90 and 150^°^C) pressurized extractions. As represented in the chromatograms, the temperature did not promote the differences in the metabolite profile. Compounds 1 and 2 were identified as linolenic acid and δ-tocopherol, respectively. In *n*-heptane chromatograms, higher intensity of δ-tocopherol was achieved, and in contrast, the peak related to linolenic acid was more intense in EM chromatograms. The peaks marked with (*) were related to phthalate contaminants identified in NaHDES candidate extracts. Further analysis should be employed focused on replacing *n*-heptane (hexane) to defat soybeans as proposed by Claux et al. (15). The analysis of fatty acid profile, neutral lipids, phospholipids, and unsaponifiable compounds will comprehensively determine the potential application of NaHDES candidates in the defatting process.

### Pressurized liquid extraction second step—Extraction of phenolic compounds

The total flavonoid (TFC) and phenolic (TPC) content of the mix of soy by-products were set as the response to optimize the pressurized conditions to extract the individual parts of soy and soybeans, as presented hereafter. First, PLE was performed using three temperatures: 60, 90, and 120^°^C; two solvents: (i) EtOH-H_2_O 7:3 (v/v) and the NADES (ii) choline chloride/citric acid/water (1:1:11) (ChCl:Ca:H_2_O); 20 min of extraction was set since it achieved the best result in the first step of PLE (Supplementary Figure 3). Such NADES was selected as its constituents and ratio have already been optimized to extract isoflavones and solubilize flavonoids under low-pressure conditions (47, 48). Bajkacz and Adamek determined the best conditions to extract daidzin, genistin, genistein, and daidzein from soy products with choline chloride/citric acid (molar ratio of 1:1 with 30% of water) at 60^°^C in ultrasound-assisted extraction (UAE) (48). Tang et al. found that a similar NADES ratio and constituents, with a slight modification in water content (37%, which means ChCl:Ca:H_2_O - 1:1:11), presents a higher capacity to extract, separate, or purify flavonoids, such as phloretin, phlorizin, and naringin DC (47). Our work selected the NADES ratio with 37% of water because it provides a lower viscosity solvent, enabling their pressurization by the PLE pump. Moreover, the TPC and TFC of PLE extractions were compared with dynamic maceration using (i) ChCl:Ca:H_2_O (at the same ratio as above) and (ii) EtOH-H_2_O 7:3 (v/v) at 60^°^C as the reference method. Additionally, GXLs with ChCl:Ca:H_2_O were tested with three CO_2_ percentages (25, 50, and 75%) at 60^°^C to reduce the amount of NADES to be employed while favoring viscosity and diffusivity of the compounds in the solvent. Mechanical problems to maintain the pressure at lower and higher CO_2_ percentages determined the utilization of just 50% in the viscous NADES. To the best of our knowledge, the literature does not report the combination of GXLs with NADES. Then, an updated GXLs equipment could be proposed as a potential innovation to deal with the solvents with a higher viscosity since, as mentioned, it could decrease their viscosity, modify the pH of the extraction media, and change other chemical and/or physical properties (24).

The results of TFC and TPC of pressurized ChCl:Ca:H_2_O and EtOH-H_2_O 7:3 (v/v), as well as GXLs and dynamic maceration extractions, are displayed in Table 3. Pressurized ChCl:Ca:H_2_O at 90 and 120^°^C and pressurized EtOH-H_2_O 7:3 (v/v) at 120^°^C presented similar results (*p* > 0.05). However, the highest average of TFC and TPC was achieved at 120^°^C and ChCl:Ca:H_2_O, which represents 369.74 ± 8.78 μg QE/g and 5.63 ± 1.11 mg GAE/g, respectively. The lowest TFC and TPC were achieved at 60°C by dynamic maceration with EtOH-H_2_O 7:3 (v/v), 136.30 ± 23.8 μg QE/g, and gas-expanded ChCl:Ca:H_2_O, which content was not detected, respectively. Aboushanab et al. extracted isoflavones from kudzu roots and soy molasses using choline chloride and citric acid at a 1:2 molar ratio (68). They achieved the TPC and TFC of 15.20 ± 3.47 mg GAE/g and 6.12 ± 0.51 mg QE/g, respectively, in soy molasses extracts. The comparison between the quoted results with our work is inadequate since they washed three times their NADES extracts with ethyl acetate 1:3 (v/v) ratio and concentrated such fractions. The dried extracts were used to perform the assays, promoting a higher concentration of TPC and TFC (68). Combining pressurized hot water extraction (PHWE) and NADES as modifiers, Loarce et al. achieved a promising approach to extract anthocyanins from grape pomace (22). In total, eight NADES mixtures were tested, and the best NADES composition was added at three percentages in PHWE (10, 20, and 30%). Subsequently, the best composition was studied at four temperatures (40, 60, 80, and 100^°^C). A PHWE with 30% choline chloride/oxalic acid 1:1 (molar ratio) resulted in the best results. In their work, higher concentrations of NADES demonstrated a higher content of anthocyanin and pyroanthocyanins. In contrast, extractions made at 60, 80, and 100^°^C did not present significant differences (*p* > 0.05) in quoted responses. Rachmaniah et al. applied pressurized NADES to extract alkaloids from *Narcissus pseudonarcissus*. The best results were accomplished using malic acid/sucrose/water (1:1:5) at 50 bar and 50^°^C, with similar results to the exhaustive Soxhlet extraction with methanol (69). Moreover, Benvenutti et al. performed pressurized DES extractions of Brazilian berry processing by-product resulting in fractions with potential antioxidant, anti-diabetic, and anti-obesity properties (23). DES solution of choline chloride with propylene glycol or malic acid combined with PLE promoted extracts with yields 50% higher than water, acidified water (pH 1.5), and hydroethanolic solution (47% of EtOH in water), which represent the conventional solvents for anthocyanins extraction.

**TABLE 3:** Total flavonoids (TFC) and phenolic content (TPC) of the mix of agro-soy by-products (i.e., branches, leaves, pods, and roots) from different extraction conditions.

Extracts	T (^°^C)	TFC *(μ g QE/g)*	TPC *(mg GAE/g)*
GXLs - ChCl:Ca:H_2_O (1:1:11)	60	136.30 ± 23.8 ^f^	^¥^
Mac - ChCl:Ca:H_2_O (1:1:11)	60	216.10 ± 40.7 ^c,d^	0.82 ± 0.13 ^e^
Mac - EtOH-H_2_O 7:3 (v/v)	60	114.21 ± 2.90 ^f^	1.83 ± 0.21 ^c,d,e^
PLE - ChCl:Ca:H_2_O (1:1:11)	60	273.8 ± 26.1 ^b,c^	1.39 ± 0.23 ^d,e^
	90	323.57 ± 16.68 ^a,b^	4.29 ± 1.15 ^a,b^
	120	369.74 ± 8.78 ^a^	5.63 ± 1.11 ^a^
PLE - EtOH-H_2_O 7:3 (v/v)	60	147.75 ± 13.93 ^e,f^	2.52 ± 0.16 ^c,d^
	90	201.39 ± 8.14 ^d,e^	3.47 ± 0.09 ^b,c^
	120	316.68 ± 13.77 ^a,b^	5.33 ± 0.13 ^a^

Letters in a column indicate significant differences between extraction solvents for different extracts (p < 0.05) by Tukey’s test. ^¥^Quantity not detected.

### Analysis of the individual parts of soy by-products and soybean

The highest TPC and TFC for the extracts prepared from the mix of soy by-products were achieved using PLE at 120^°^C with ChCl:Ca:H_2_O. Then, this condition was applied to extract soy by-products individually and soybeans (previously defatted by pressurized EM); results are presented in Table 4. Extracts of soy leaves followed by pods presented the higher TFC, 419.10 ± 33.8 and 250.10 ± 20.00 μg QE/g, respectively. Soy branches, roots, and soybeans did not present a significant difference (*p* > 0.05) in TFC. For soy by-products, the average value of TPC was 8.45 ± 2.26 mg GAE/g, with no difference among them. However, soybeans contained the lowest TPC value, 1.01 ± 0.18 mg GAE/g. Comparisons between the content of bioactive compounds, specifically isoflavones, of soy by-products and soybeans were reported by Carneiro et al. (10). The authors found that soy by-products have 132% of the total isoflavones quantified in the soybeans, representing that soy by-products are potential sources of phenolic compounds. In addition, Cabezudo et al. achieved a higher content of phenolic compounds in soy hull, a soy by-product, than in soybeans (70). They performed a green extraction based on an alkaline hydrolysis treatment, resulting in a TPC of 0.72 g gallic acid equivalents per 100 g of soybean hull. Gupta and Chen promoted a higher content of daidzein and genistein in fermented okara (soymilk by-product) extracts using *Rhizopus oligosporus* compared to the unfermented material (71). Moreover, the fermented extracts presented a TPC and TFC of 709.33 ± 2.92 and 10.77 ± 1.25 mg of gallic acid and quercetin equivalents per g of okara samples, respectively. Regarding the antioxidant properties of the soy by-products and commodity extracts, ORAC was determined by ROS scavenging capacity method (Table 4). Soy root extracts presented the highest antioxidant capacity, 207.48 ± 40.23 mg AA/g, with three times more activity than soybeans extracts, 68.96 ± 12.30. Leaves and branches contained similar antioxidant capacity, with 124.75 ± 12.71 and 108.33 ± 20.61 mg AA/g. In contrast, pods had the lowest value, 60.74 ± 13.60 mg AA/g. Dorta et al. compared the TPC and ORAC of mango by-products, concluding that both responses did not present a correlation (72). In contrast, applying a combination of PLE and NADES, Benvenutti et al. found a correlation between TPC and antioxidant capacity of Brazilian berry processing by-product extracts (23). In addition, intermediate DES concentrations, flow rate, and temperature increased the antioxidant potential of the extracts. Performing subcritical water with NADES, Loarce et al. accomplished extracts with a higher content of catechins, tannins, hydroxycinnamic acids, and flavonols from winemaking by-products (21). Besides, they referred that quoted bioactive metabolites contribute to antioxidant capacity. According to the best of our knowledge, the literature does not report the combination of PLE and NADES to generate phenolic-rich fractions with high antioxidant capacity from agro-soy by-products, which have been described as the promising sources of bioactive compounds and sparsely explored (11).

**TABLE 4:** Total content of flavonoids (TFC) and phenolics (TPC), as well as antioxidant capacity (ORAC) of the individual extracts of soy by-products (i.e., branches, leaves, pods, and roots) and soybeans resulted from pressurized choline chloride/citric acid/water (1:1:11) at 120^°^C, 100 bar, and 20 min of extraction.

Extracts	TFC (μ g QE/g)	TPC (mg GAE/g)	ORAC (mg AA/g)
Branches	154.00 ± 21.8 ^c^	6.35 ± 1.67 ^a^	108.33 ± 20.61 ^b,c^
Leaves	419.10 ± 33.8 ^a^	9.73 ± 1.27 ^a^	124.75 ± 12.71 ^b^
Pods	250.10 ± 20.00 ^b^	9.28 ± 2.16 ^a^	60.74 ± 13.60 ^c^
Roots	114.37 ± 14.68 ^c^	8.44 ± 2.99 ^a^	207.48 ± 40.23 ^a^
Soybeans^¥^	154.00 ± 47.10 ^c^	1.01 ± 0.18 ^b^	68.96 ± 12.30 ^b,c^

Letters in a column indicate significant differences between extraction solvents for different extracts (p < 0.05) by Tukey’s test. ^¥^Defatted soybeans.

Pressurized ChCl:Ca:H_2_O extracts were also analyzed by UHPLC-ESI-QToF-MS/MS. The annotation of the MS/MS spectra was performed by comparing its data against the GNPS spectral reference library and manually checking by MZmine (54). Additionally, the candidates obtained by GNPS had their compatibility challenged with the acquired high-resolution mass and with previous reports of their occurrence in soy by-products as organized in a database published elsewhere (5). Table 5 summarizes the annotation of identified metabolites, focusing on the most important metabolites in soy, flavonoids, and isoflavonoids. Isoflavonoids such as daidzin and genistin, as well as flavonoid apigenin, were found in all soy parts. Such metabolites were related to potential bioactive properties against cancer and anti-inflammatory capacity (7, 73–75). Moreover, bioactive isoflavonoids, such as formononetin 7-O-glucoside (ononin) and biochanin A 7-O-glucoside (astroside), were identified in at least one soy by-product. Ononin has been related as an anti-parasitic drug, and astroside contains potential properties to maximize the microbial community function in animals (76, 77).

**TABLE 5:** List of tentatively identified compounds in the pressurized choline chloride/citric acid/water (1:1:11) extracts of soy branches (B), leaves (L), pods (P), roots (R), and soybeans (S) by UHPLC-ESI-QToF-MS/MS.

Peak number	Rt (min)	Tentative identification	Classification	Molecular formula	Molecular Ion	Measured mass (Δ ppm)	MS/MS fragments (relative abundance)	B	L	P	R	S	Ref.
1	4.18	Licoagroside B	Glycoside	C18H24O12	[M+H]+	433.1340 (3.8)	127.0380 (100), 85.0280 (7.8), 128.0420 (6.9)	X	X	X	X	X	
2	5.06	Genistin	Isoflavonoids	C21H20O10	[M+H]+	433.1129 (-3.9)	255.0650 (100), 256.0680 (19.1), 199.0740 (9.1)	X	X	X	X	X	
3	5.26	Daidzin	Isoflavonoids	C21H20O9	[M+H]+	417.1180 (-0.4)	255.0670 (100), 256.0660 (25.2), 257.0700 (6.3)	X	X	X	X	X	
					[M+FA-H]-	461.1089 (-4.5)	253.0490 (100), 252.0420 (61.1), 44.9990 (56.7)						
4	5.30	Kaempferol 3-rutinoside 4′-glucoside	Flavonoids	C33H40O20	[M+H]+	757.2186 (6.6)	287.0540 (100), 85.0270 (28.6), 288.0570 (21.3)		X	X			
5	5.36	3-{[(2S,3R,4S,5S,6R)-4,5-Dihydroxy-3-{[3,4,5-trihydroxy-6-(hydroxymethyl) oxan-2-yl]oxy}-6-({[(2R,3R,4R,5R,6S)-3,4,5-trihydroxy-6-methyloxan-2-yl]oxy}methyl) oxan-2-yl]oxy}-5,7-dihydroxy-2-(4-hydroxyphenyl) chromen-4-one	Flavonoids	C33H40O20	[M+H]+	757.2185 (3.8)	287.0550 (100), 288.0590 (20.1), 85.0270 (15.2)		X		X		
6	5.45	Biochanin A 7-O-glucoside (astroside)	Isoflavonoids	C22H22O10	[M+Cl]-	481.0907 (-1.7)	283.0570 (100), 268.0370 (38.0), 284.0620 (17.2)			X		X	
7	5.56	Glycitin	Isoflavonoids	C22H22O10	[M+H]+	447.1286 (0.2)	285.0746 (100), 286.0786 (21.8), 448.2425 (8.6)	X	X	X		X	
8	6.50	Malonyldaidzin	Isoflavonoids	C24H22O12	[M+H]+	503.1184 (4.2)	255.0647 (100), 70.0657 (20.4), 86.0949 (20.4)			X	X	X	
9	7.10	6?-O-Acetyldaidzin	Isoflavonoids	C23H22O10	[M+H]+	459.1286 (3.9)	255.0649 (100), 70.0658 (12.0), 471.2798 (10.1)					X	
10	7.26	Formononetin 7-O-glucoside (ononin)	Isoflavonoids	C22H22O9	[M+H]+	431.1336 (6.9)	269.0800 (100), 270.0830 (25.6), 254.0560 (11.6)	X	X	X			
11	7.41	4′,6-Dimethoxyisoflavone-7-O-.beta.-D-glucopyranoside (wistin)	Isoflavonoids	C23H24O10	[M+H]+	461.1442 (2.9)	299.0900 (100), 284.0670 (42.6), 300.0930 (19.0)		X				
12	8.45	Apigenin	Flavonoids	C15H10O5	[M-H]-	269.0455 (-5.8)	269.0412 (100), 151.0321 (23.4), 223.8405 (21.9)	X	X	X	X	X	

Despite the high temperature, 120^°^C, which could affect the glucoside forms of the flavonoids and isoflavonoids, 6 -O-acetyldaidzin, malonyldaidzin, daidzin, genistin, glycitin, and kaempferol 3-rutinoside 4′-glucoside were also identified in the extracts. Rostagno et al. found that the degradation of malonyl-glucoside and glucosides occur over 100 and 150^°^C, respectively (78). Therefore, NADES composition may have interfered with the temperature effect on flavonoids and isoflavonoids. Nevertheless, PLE-NADES-based extracts were diluted before the injection in the MS systems as a precautionary step, which could have decreased the identification of minority compounds. Alternative methodologies for recovery of phenolic compounds from NADES extracts, such as liquid–liquid extraction (LLE), solid-phase extraction (SPE), and supercritical carbon dioxide extraction (SFC), may be feasible approaches to concentrate target metabolites and reduce the interference of NADES components, as mentioned by Mišan et al. (79).

## Conclusion

PLE using choline chloride/citric acid/water (1:1:11—molar ratio) at 120^°^C, 100 bar, and 20 min (2 cycles of 10 min) resulted in an optimized conditions to extract phenolics and flavonoids from agro-soy by-products. Extracts of soy leaves obtained under these conditions contained the highest TFC, followed by pods, with 419.10 ± 33.8 and 250.10 ± 20.00 μg QE/g, respectively. Regarding TPC, soy by-products contain similar content and are higher than soybeans, whereas soy root extracts presented the highest antioxidant capacity, 207.48 ± 40.23 mg AA/g. This was three times higher than that observed for soybeans extracts, with 68.96 ± 12.30 mg AA/g. Moreover, bioactive compounds, such as apigenin (flavonoid) and daidzin and genistin (isoflavonoids), were identified in PLE-NADES-based extracts of all parts of soy. Additionally, a new defatting process was developed using pressurized NaHDES candidates, with a similar yield and chromatography profile compared to *n*-heptane, becoming a new and greener alternative to conventional soybean oil extraction.

This work promotes new insights into the use of pressurized and gas-expanded NADES, which have been sparsely combined and present promising applications. Such solvents can be green alternatives to extract bioactive compounds and, combined with pressurized techniques, could be a powerful approach to generate phenolic-rich fractions with human health benefits. In addition, the use of an abundant agricultural by-product, i.e., soy branches, leaves, pods, and roots, which contains several bioactive compounds, is related to a circular and greener economy, as suggested by the goal 12, “Sustainable consumption and production” of the 17 sustainable development goals (SDGs) set by the United Nations (UN).

## Data availability statement

The original contributions presented in this study are included in the article/Supplementary material, further inquiries can be directed to the corresponding author.

## Author contributions

FB, BS-R, JM, AC, CF, and EI: conceptualization and methodology. FB, CF, and EI: writing – original draft preparation, review, and editing. FB: software and data curation. All authors have read and agreed to the published version of the manuscript.

## Footnotes

1
https://www.chemaxon.com

2
http://gnps.ucsd.edu

3
https://ccms-ucsd.github.io/GNPSDocumentation/

4
https://mzmine.github.io/
