# Supplementary material for: Pressurized natural deep eutectic solvents: An alternative approach to agro-soy by-products
Source: Front Nutr. 2022 Sep 8;9:953169. doi: 10.3389/fnut.2022.953169 (PMC9493435; doi:10.3389/fnut.2022.953169)
Supplement: Supplementary file 1 [file Data_Sheet_1.pdf]

Supplementary Material

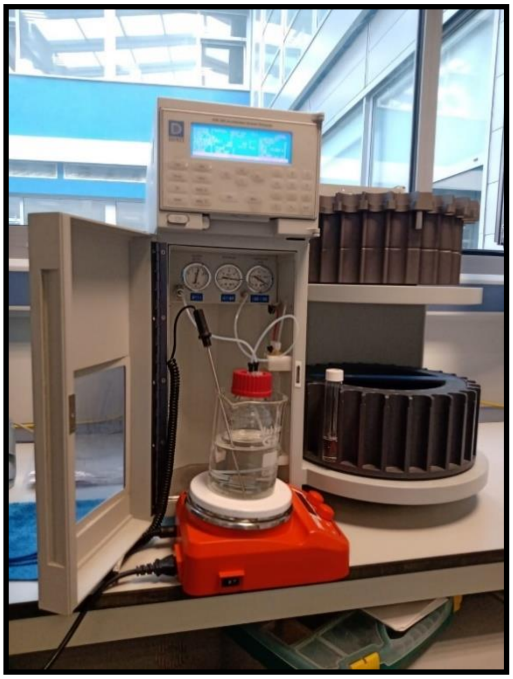

Figure S1. Equipment used to perform the pressurized Natural Hydrophobic Eutectic Solvents (NaHDES) extraction (ASE 200 device - Dionex, USA), with the bottle of respective solvents maintained at 90 °C.

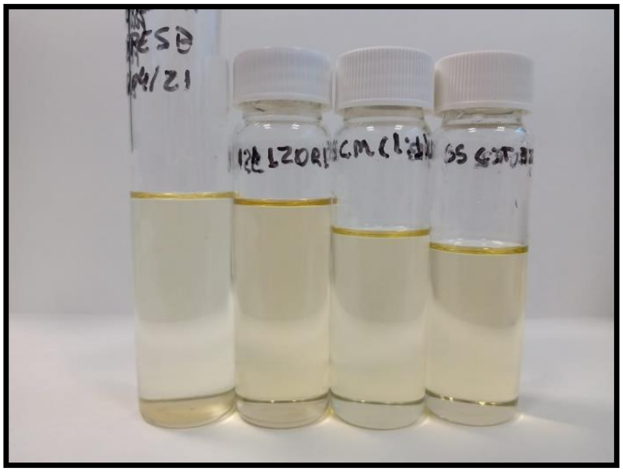

Figure S2. Pressurized *n*-heptane and Natural Hydrophobic Eutectic Solvents (NaHDES) extracts of soybean at 120 °C. As follows: *n*-heptane, eucalyptol:menthol (1:1), camphor:menthol (1:1), camphor:thymol (3:2) extracts.

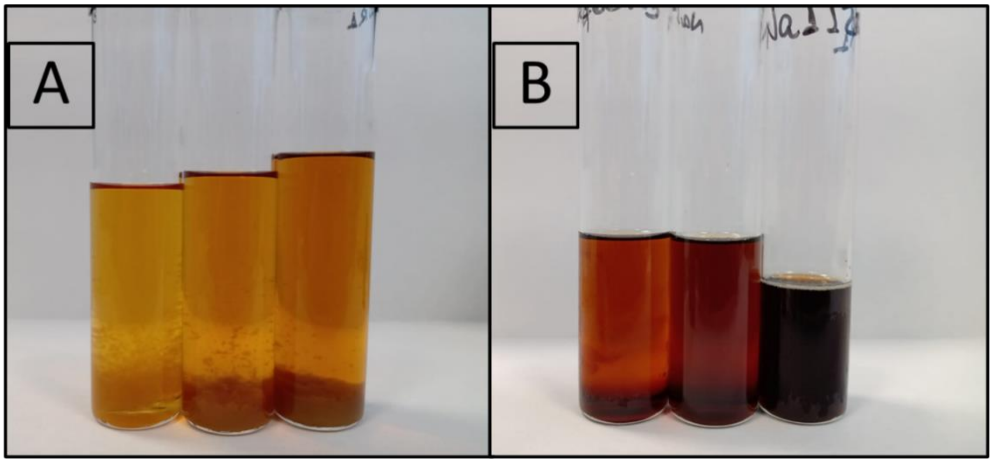

Figure S3. Pressurized EtOH-H2O 7:3 (v/v) (A) and Natural Eutectic Solvents (NADES) (B) extracts of the mix of soy parts, i.e., branches, leaves, pods, and roots, at 60, 90, and 120 °C, respectively.

Table S1. Natural Hydrophobic Eutectic Solvents (NaHDES) (1–18) with their respective Hansen Solubility Parameters (HSP) and distance (Ra) compared to *n*-heptane. A lower Ra value represents a higher similarity between the NaHDES and *n*-heptane.

Nahdes (molar ratio)	Dtotal	Ptotal	Htotal	Ra	Ref
Heptane	*15.30*	*0.00*	*0.00*	*0.00*	
**Menthol:Eucalyptol(1:1)***	**16.59**	**3.17**	**4.82**	**6.33**	**18**
Borneol:Oleic acid(1:4)	16.46	3.08	5.71	6.88	15
Borneol:Oleic acid(1:3)	16.47	3.10	5.76	6.95	15
Borneol:Oleic acid(1:2)	16.50	3.14	5.87	7.08	15
**Camphor:Menthol(1:1)***	**16.91**	**4.28**	**4.71**	**7.13**	**10,18**
Menthol:Camphor(2:1)	16.77	4.00	5.48	7.39	5,10
Camphor:Thymol(2:1)	17.53	4.80	3.81	7.58	10,13
**Camphor:Thymol(3:2)***	**17.56**	**4.72**	**4.16**	**7.74**	**13**
Camphor:Thymol(1:1)	17.60	4.60	4.67	8.01	4,5,10,13
Menthol:Myristic acid(8:1)	16.47	3.47	6.86	8.03	18
Menthol:Dodecanoic acid(2:1)	16.42	3.69	7.13	8.33	3,7,11
Perillyl alcohol:Camphor(1:1)	17.35	4.74	5.70	8.47	18
Camphor:Thymol(1:2)	17.67	4.40	5.51	8.49	10,13
Thymol:Menthol(1:2)	16.89	3.65	6.99	8.50	1,10,17
1-Tetradecanol:Menthol(1:2)	16.34	3.38	7.68	8.64	1
Decanoic acid:Menthol(1:2)	16.43	3.74	7.48	8.66	1,5
Thymol:Menthol(1:1)	17.10	3.73	7.04	8.74	1,5,8,10,17
Menthol:Decanoic acid(3:2)	16.42	3.79	7.59	8.77	5,8
Nonanoic acid:Menthol(1:1,5)	16.42	3.85	7.75	8.94	8
Decanoic acid:Menthol(1:1)	16.40	3.86	7.76	8.94	1,5,7,9,11
Menthol:Thymol(1:2)	17.32	3.82	7.09	9.01	10,17
Borneol:Thymol(1:1)	17.34	3.85	7.25	9.17	15
Menthol:Lidocaine(2:1)	17.04	5.31	6.63	9.18	1

Borneol:Thymol(1:2)	17.49	3.90	7.23	9.31	15
Decanoic acid:Palmitic acid(8:1)	16.28	4.06	8.15	9.32	7
Decanoic acid:Myristic acid(5:1)	16.30	4.01	8.18	9.32	7
Octanoic acid:Menthol(1:1,5)	16.43	4.12	8.06	9.33	8
Decanoic acid:dodecanoic acid(2:1)	16.30	4.13	8.19	9.39	2,4,9
Borneol:thymol(1:3)	17.57	3.92	7.23	9.39	15
Menthol:Perillyl alcohol(1:1)	16.87	3.87	7.97	9.40	18
Dodecanoic acid:lidocaine(2:1)	16.84	5.43	7.06	9.42	1
Borneol:thymol(1:4)	17.62	3.94	7.22	9.44	15
Dodecanoic acid:Atropine(2:1)	17.06	4.14	7.72	9.44	1
Borneol:Decanoic acid(1:2)	16.48	4.05	8.21	9.45	15
Decanoic acid:Dodecanoic acid(3:1)	16.30	4.14	8.29	9.48	7
C9:C10:C12 (1:1:1)	16.30	4.18	8.33	9.53	2
Borneol:Decanoic acid(1:3)	16.43	4.09	8.31	9.54	15
Decanoic acid:Thymol(1:1)	16.98	4.11	7.97	9.57	4,5,8,9
Borneol:Decanoic acid(1:4)	16.41	4.11	8.37	9.59	15
C9:C10:C12 (1:2:1)	16.30	4.19	8.40	9.59	2
Menthol:Butyric acid(1:1)	16.20	4.03	8.53	9.60	11
Menthol:Acetic acid(1:1)	16.19	4.23	8.48	9.64	3,11,16
Menthol:Octanoic acid(1:1)	16.41	4.28	8.38	9.67	9,11
C9:C10:C12 (2:1:1)	16.30	4.23	8.51	9.71	2
C9:C10:C12 (2:2:1)	16.30	4.23	8.53	9.72	2
C9:C10:C12 (3:2:1)	16.30	4.25	8.61	9.81	2
C9:C10:C12 (3:1:1)	16.30	4.26	8.62	9.82	2
Nonanoic acid:Dodecanoic acid(3:1)	16.30	4.28	8.62	9.83	2
Nonanoic acid:Thymol(1:1)	17.01	4.21	8.20	9.83	8
C8:C10:C12 (1:1:1)	16.30	4.40	8.58	9.85	2
C8:C9:C12 (1:1:1)	16.30	4.47	8.74	10.02	2

C8:C9:C12 (1:2:1)	16.30	4.45	8.83	10.09	2
Decanoic acid:Lidocaine(2:1)	16.90	5.71	7.70	10.10	1
1-Naphthol:Menthol(1:2)	17.31	4.21	8.27	10.11	1
Decanoic acid:Atropine(2:1)	17.14	4.28	8.40	10.12	1
Menthol:Anisyl alcohol(2:1)	17.18	4.60	8.28	10.19	12
C8:C10:C12 (2:1:1)	16.30	4.57	8.91	10.21	2
Thymol:Lidocaine(2:1)	17.84	5.78	6.78	10.25	1
Atropine:Thymol(1:2)	18.07	4.18	7.60	10.29	1
Menthol:Hexanoic acid(1:1)	16.42	4.43	9.04	10.32	11
C8:C10:C12 (3:2:1)	16.30	4.60	9.03	10.33	2
C8:C9:C12 (2:1:1)	16.30	4.63	9.04	10.35	2
Thymol:Octanoic acid(1:1)	17.05	4.60	8.65	10.41	9
C8:C10:C12 (3:1:1)	16.30	4.68	9.12	10.45	2
C8:C9:C12 (3:2:1)	16.30	4.68	9.21	10.52	2
Decanol:Thymol(1,5:1)	16.71	3.94	9.39	10.56	8
Octanoic acid:Thymol(29:21)	16.93	4.70	8.89	10.57	8
C8:C9:C12 (3:1:1)	16.30	4.73	9.23	10.57	2
Thymol:Lidocaine(1:1)	17.86	6.50	6.61	10.58	1
Thymol:Coumarin(2:1)	18.22	5.81	6.73	10.63	1,10
Octanoic acid:Dodecanoic acid(3:1)	16.30	4.82	9.27	10.63	2,9
Dodecanoic acid:Butyric acid(1:3)	15.91	4.61	9.87	10.96	4
Thymol:Coumarin(1:1)	18.45	6.83	6.46	11.32	1,5,10
Menthol:Anisyl alcohol(1:1)	17.58	5.26	9.10	11.46	12
Menthol:Levulinic acid(1:1)	16.79	5.99	9.39	11.53	11
Octanol:Thymol(2:1)	16.67	4.27	10.60	11.75	8
1,2-Decanediol:Thymol(1:2)	17.27	4.53	10.14	11.78	1
Dodecanoic acid:Hexanoic acid(1:3)	16.30	5.13	10.45	11.81	4
Coumarin:Thymol(2:1)	18.71	7.94	6.17	12.15	10
Menthol:Anisyl alcohol(1:2)	18.04	6.02	10.05	12.93	12

Menthol:Lactic acid(1:1)	16.78	5.66	11.30	12.98	5
Imidazole:Acetic acid(1:2)	16.85	8.77	13.01	13.12	14
Imidazole:Octanoic acid(1:2)	16.89	6.46	10.43	13.16	14
Imidazole:Butyric acid(1:2)	16.69	7.06	11.85	13.32	14
Menthol:Acetic acid(1:2)	16.00	4.68	9.46	13.51	14
Pyrazole:Octanoic acid(1:2)	16.97	6.10	10.50	13.54	14
Menthol:Octanoic acid(1:2)	16.37	4.57	8.92	13.64	14
Pyrazole:Decanoic acid(1:2)	16.88	5.12	9.16	13.66	14
Imidazole:Hexanoic acid(1:2)	17.01	7.20	12.16	13.69	14
Menthol:Butyric acid(1:2)	16.05	4.30	9.34	13.83	14
Pyrazole:Acetic acid(1:2)	17.04	7.99	13.16	13.83	14
Menthol:Hexanoic acid(1:2)	16.38	4.82	9.95	13.85	14
Pyrazole:Butyric acid(1:2)	16.82	6.51	11.96	13.89	14
Menthol:Pyrazole(3:1)	16.89	4.24	7.49	13.89	14
Benzyl alcohol:Thymol(4:1)	18.67	5.45	11.03	14.03	8
Pyrazole:Hexanoic acid(1:2)	17.11	6.76	12.24	14.12	14
Menthol:Pyruvic acid(1:2)	17.07	7.71	11.33	14.15	3,11
Thymol:Lactic acid(1:1)	17.70	6.25	12.02	14.38	5
Menthol:lactic acid(1:2)	16.94	6.86	13.75	15.72	3
Leucine:Lactic acid(1:3)	17.01	8.58	18.76	20.91	6
Arginine:Lactic acid(1:3)	17.23	10.51	18.37	21.52	6
Leucine:Lactic acid(1:4)	17.10	9.02	19.38	21.68	6
Arginine:Lactic acid(1:4)	17.27	10.61	19.05	22.16	6
Leucine:Lactic acid(1:5)	17.16	9.32	19.83	22.22	6
Arginine:Lactic acid(1:5)	17.31	10.68	19.54	22.62	6
Serine:Lactic acid(1:5)	17.54	11.21	22.74	25.74	6
Serine:Lactic acid(1:4)	17.54	11.23	22.80	25.81	6
Serine:Lactic acid(1:3)	17.55	11.26	22.90	25.91	6

***NaHDES selected to defat soybeans using PLE**

REFERENCES

1. van Osch DJGPGP, Dietz CHJTJT, van Spronsen J, Kroon MC, Gallucci F, van Sint Annaland M, et al. A Search for Natural Hydrophobic Deep Eutectic Solvents Based on Natural Components. ACS Sustainable Chemistry and Engineering [Internet]. 2019 Mar 4 [cited 2021 Nov 24];7(3):2933–42. Available from: https://pubs.acs.org/sharingguidelines

2. Florindo C, Romero L, Rintoul I, Branco LC, Marrucho IM. From Phase Change Materials to Green Solvents: Hydrophobic Low Viscous Fatty Acid-Based Deep Eutectic Solvents. ACS Sustainable Chemistry and Engineering [Internet]. 2018 Feb 4;6(3):3888–95. Available from: https://doi.org/10.1021/acssuschemeng.7b04235

3. Ribeiro BD, Florindo C, Iff LC, Coelho MAZ, Marrucho IM. Menthol-based eutectic mixtures: Hydrophobic low viscosity solvents. ACS Sustainable Chemistry and Engineering [Internet]. 2015 Mar 4;3(10):2469–77. Available from: https://doi.org/10.1021/acssuschemeng.5b00532

4. Morelli DC, Bernardi G, Morés L, Pierri ME, Carasek E. A green - high throughput – extraction method based on hydrophobic natural deep eutectic solvent for the determination of emerging contaminants in water by high performance liquid chromatography – diode array detection. Journal of Chromatography A [Internet]. 2020 Mar 4;1626:461377. Available from: https://www.sciencedirect.com/science/article/pii/S0021967320306543

5. Ye X, Ye B, Xu J, Fang M, Dong D, Wu C, et al. A modified quick, easy, cheap, effective, rugged, and safe method with hydrophobic natural deep eutectic solvent as extractant and analyte protectant for analyzing pyrethroid residues in tomatoes. Journal of Separation Science [Internet]. 2020 Mar 4;43(17):3546–54. Available from: https://onlinelibrary.wiley.com/doi/abs/10.1002/jssc.202000547

6. Nia NN, Hadjmohammadi MR. Amino acids- based hydrophobic natural deep eutectic solvents as a green acceptor phase in two-phase hollow fiber-liquid microextraction for the determination of caffeic acid in coffee, green tea, and tomato samples. Microchemical Journal [Internet]. 2021 Mar 4;164:106021. Available from: https://www.sciencedirect.com/science/article/pii/S0026265X21001065

7. Haider MB, Dwivedi M, Jha D, Kumar R, Marriyappan Sivagnanam B. Azeotropic separation of isopropanol-water using natural hydrophobic deep eutectic solvents. Journal of Environmental Chemical Engineering [Internet]. 2021 Feb 28;9(1):104786. Available from: https://www.sciencedirect.com/science/article/pii/S2213343720311350

8. Sereshti H, Semnani Jazani S, Nouri N, Shams G. Dispersive liquid–liquid microextraction based on hydrophobic deep eutectic solvents: Application for tetracyclines monitoring in milk. Microchemical Journal [Internet]. 2020 Mar 5;158:105269. Available from: https://www.sciencedirect.com/science/article/pii/S0026265X20316568

9. Sas OG, Castro M, Domínguez Á, González B. Removing phenolic pollutants using Deep Eutectic Solvents. Separation and Purification Technology [Internet]. 2019 Mar 5;227:115703. Available from: https://www.sciencedirect.com/science/article/pii/S1383586619317988

10. Hansen FA, Santigosa-Murillo E, Ramos-Payán M, Muñoz M, Leere Øiestad E, Pedersen-Bjergaard S. Electromembrane extraction using deep eutectic solvents as the liquid membrane. Analytica Chimica Acta [Internet]. 2021 Mar 4;1143:109–16. Available from: https://www.sciencedirect.com/science/article/pii/S0003267020311740

11. Florindo C, Branco LC, Marrucho IM. Development of hydrophobic deep eutectic solvents for extraction of pesticides from aqueous environments. Fluid Phase Equilibria [Internet]. 2017 Mar 4;448:135–42. Available from: https://www.sciencedirect.com/science/article/pii/S0378381217301425

12. Fan Y, Wu H, Cai D, Yang T, Yang L. Effective extraction of harmine by menthol/anise alcohol-based natural deep eutectic solvents. Separation and Purification Technology [Internet]. 2020 Mar 4;250:117211. Available from: https://www.sciencedirect.com/science/article/pii/S1383586620316853

13. Li Y, Li X, Tang S, Yang Y. Emulsification liquid–liquid micro-extraction based on natural deep eutectic solvent for (triarylmethane) dyes determination. Chemical Papers [Internet]. 2020 Mar 4;74(10):3617–26. Available from: https://doi.org/10.1007/s11696-020-01184-4

14. Lo C, Semerel J, van den Berg C, Wijffels RH, Eppink MHM. Eutectic solvents with tuneable hydrophobicity: lipid dissolution and recovery. RSC Advances [Internet]. 2021 Feb 25;11(14):8142–9. Available from: https://pubs.rsc.org/en/content/articlelanding/2021/ra/d1ra00306b

15. Majidi SM, Hadjmohammadi MR. Hydrophobic borneol-based natural deep eutectic solvents as a green extraction media for air-assisted liquid-liquid micro-extraction of warfarin in biological samples. Journal of Chromatography A [Internet]. 2020 Mar 4;1621:461030. Available from: https://www.sciencedirect.com/science/article/pii/S0021967320302466

16. Ortega-Zamora C, González-Sálamo J, González-Sálamo J, Hernández-Sánchez C, Hernández-Sánchez C, Hernández-Borges J, et al. Menthol-Based Deep Eutectic Solvent Dispersive Liquid-Liquid Microextraction: A Simple and Quick Approach for the Analysis of Phthalic Acid Esters from Water and Beverage Samples. ACS Sustainable Chemistry and Engineering [Internet]. 2020 Mar 4;8(23):8783–94. Available from: https://doi.org/10.1021/acssuschemeng.0c02603

17. Santana-Mayor Á, Socas-Rodríguez B, Rodríguez-Ramos R, Herrera-Herrera A v., Rodríguez-Delgado MÁ. Quality assessment of environmental water by a simple and fast non-ionic hydrophobic natural deep eutectic solvent-based extraction procedure combined with liquid chromatography tandem mass spectrometry for the determination of plastic migrants. Analytical and Bioanalytical Chemistry [Internet]. 2021 Mar 4;413(7):1967–81. Available from: https://doi.org/10.1007/s00216-021-03166-1

18. Rodrigues LA, Pereira C v., Leonardo IC, Fernández N, Gaspar FB, Silva JM, et al. Terpene-Based Natural Deep Eutectic Systems as Efficient Solvents to Recover Astaxanthin from Brown Crab Shell Residues. ACS Sustainable Chemistry and Engineering [Internet]. 2020 Mar 4;8(5):2246–59. Available from: https://doi.org/10.1021/acssuschemeng.9b06283
